# Supplementary figures and images for: Pectin Peek-a-Boo: Homogalacturonan Turnover During Flooding-Induced Legume Root Aerenchyma Formation
Source: Plants (Basel). 2025 Aug 23;14(17):2620. doi: 10.3390/plants14172620 (PMC12430375; doi:10.3390/plants14172620)

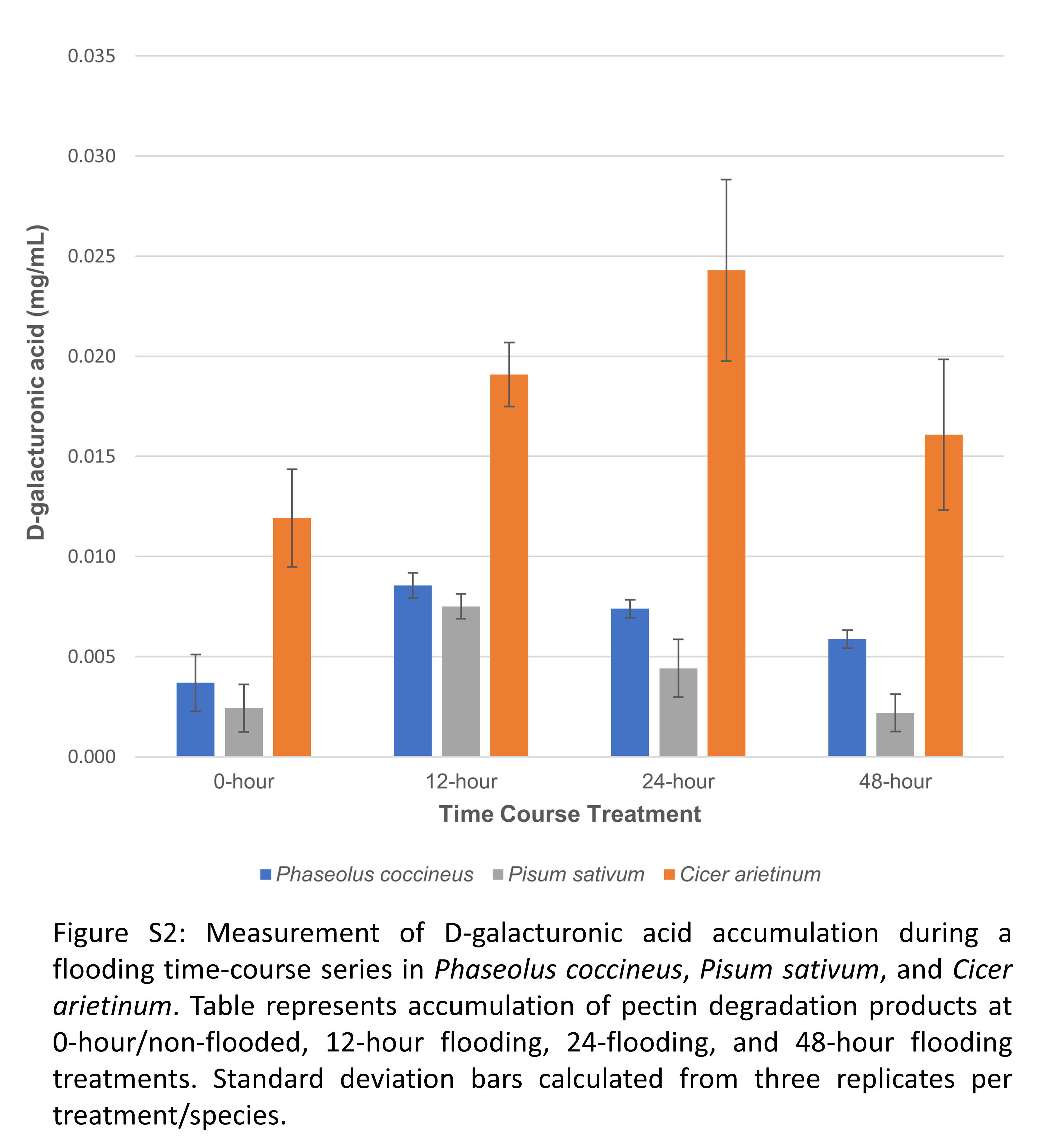

Supplement: Supplementary file 1 [file plants-14-02620-s001.zip › plants-3720894_Fig_S2.png]

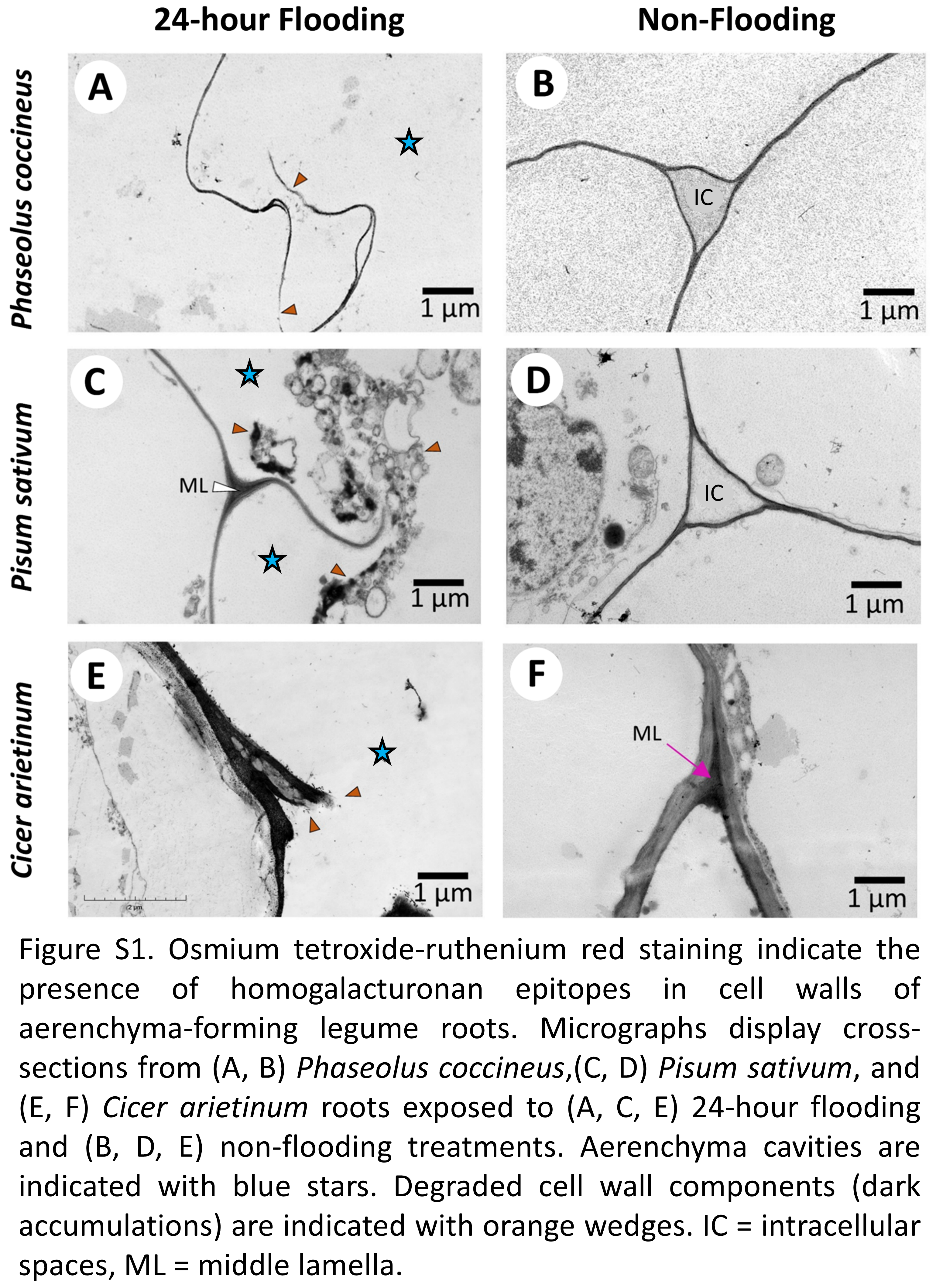

Supplement: Supplementary file 1 [file plants-14-02620-s001.zip › plants-3720894_Fig_S1.png]
